# Identifying terrorist attack victims

**DOI:** 10.1080/20961790.2020.1821149

**Published:** 2020-11-02

**Authors:** Elvire Arrighi, Amandine Matricon Charlot

**Affiliations:** a National French Police, Direction Centrale de la Police Judiciaire, Paris, France; b French DVI Police Unit, 31 avenue Franklin Rossevelt, 69130 Ecully, Lyon, France

**Keywords:** Forensic sciences, victims identification, terrorist attacks, mass killing, INTERPOL, Identification Commission, antemortem unit, postmortem unit

## Abstract

The terrorist attacks that occurred in France in 2015 and 2016, which had many victims, proved that it is essential to identify victims following the methodology developed by International Criminal Police Organization (INTERPOL) for such events. Initially designed for natural disasters, this approach must be strictly respected should a terrorist attack occur. This includes the specific collection of bodies and body parts, as well as the setup of an antemortem unit, postmortem unit, and Identification Commission. This commission is made up of specialists and will make decisions on the basis of primary identifying elements (fingerprints, DNA, dental records) and/or secondary identifying elements (other distinctive and particularly discriminating signs). A multidisciplinary team, combining specifically trained police officers and scientists, must provide a reliable and consolidated list of deceased people based on biometric elements cross checked with elements from the investigations. Any list of names generated on another basis should be excluded. Identification of the deceased by relatives (visual recognition), even if the bodies do not appear mutilated and/or decayed, should be avoided to avert erroneous death announcements, body presentations, or even burials or cremations. Similarly, identifying victims only on the basis of their personal effects (such as an identity card) must be absolutely avoided. All bodies, even those whose identities seem obvious to the first responders or to the relatives, must be registered as “X” during the crime scene search and properly identified according to the INTERPOL protocol. The same protocol should be applied to unconscious injured people. Considering the expectations of our modern society for rapid information circulation and quick responses from the authorities, the French team has made a few adjustments to speed up the identification process without compromising its integrity. Validated and supported by both the French judicial and administrative authorities in the light of the experience of the November 2015 attacks in Paris, this innovative method proved its effectiveness during the Nice attack in July 2016. It can only be successful in a context where all the individuals involved in the crisis, up to the highest authorities, understand it, support it, and relay it in the best interest of the victims’ families.

**KEY POINTS**
- The INTERPOL protocol must be used in order to identify victims after a terrorist attack.
- Some adaptations to the abovementioned protocol can be put in place in order to speed up the identification process in such circumstances.
- The lessons learned from the 2015 Paris terrorist attack can be useful to other disaster victim identification (DVI) units.

## Introduction

During the November 2015 terrorist attacks in Paris, France where 130 people were killed, the urgency of notifying families whether their next of kin was dead or alive led to partial implementation of the disaster victims identification (DVI) protocol. Both the media and the state authorities were demanding an immediate communication of the identities of the victims, with frustrated relatives still waiting to hear news and eager to be taken to their next of kin’s location. Social media did not help: through them, people conducted research by their own means, and performed unverified victim identifications. This “immediacy culture” was associated with a general ignorance and/or misunderstanding of the DVI protocol in France during this national crisis, and had severe consequences for both families of victims and government services. Because of this experience, the various individuals involved in mass murder cases in France have since applied the DVI protocol to all victims, whether they carry identification or not. This was especially the case in Nice in July 2016. The application of the DVI protocol should not prevent the implementation of measures to speed up and adapt this process to ensure both reliable identifications and early notifications to families.

Initiated by the Police Unit for DVI (Unité Police d’Identification de Victimes de Catastrophes, hereafter “DVI Police Unit”) of the Central Department of the Technical and Scientific Police (Service Central de la Police Technique et Scientifique, hereafter “SCPTS”), the “French-style” DVI methodology has been praised in various conferences. These include the annual INTERPOL conference on DVI in Lyon in 2016 and Singapore in 2017, the Australasian DVI conference in 2017, the mass fatality symposium organised by the office of the Chief Medical Examiner of Washington in 2016, and others. Within a scope validated and promoted by the Sous-Direction Anti-Terroriste (SDAT) of the Central Directorate of the Judicial Police (Direction Centrale de la Police Judiciaire, hereafter DCPJ), the DVI protocol has been adapted to situations of mass murder and the demands of a society that lives in the immediacy. This methodology is now being used internationally as a reference.

## Methodology for DVI

Initially designed for natural disaster situations, the DVI methodology is described in a guide first issued by INTERPOL in 1984 and adjusted following the 2004 tsunami in Asia. The aim is to scientifically identify large numbers of deceased individuals using a standardized methodology and a common nomenclature among all INTERPOL member countries. The DVI protocol excludes identification by relatives or by a document bearing the identity of the victim, such as an identity or transport card. Specialized units are specifically trained.

### The “antemortem unit”

The “antemortem unit” collects information regarding the physical elements of the missing person (e.g. size, weight, hair colour, tattoos, scars, medical and dental information) and other technical elements (possible papillary and genetic traces) from families (e.g. through family/friend interviews, collecting objects such as the toothbrush of the missing person). Any information collected by another unit relating to the missing person should be transmitted to the antemortem unit.

### The “postmortem unit”

The “postmortem unit” simultaneously records all materials found on the deceased victim that could be used for their identification (e.g. tattoos, ID card, personal effects). They are surveyed for papillary traces and DNA sampling. This unit, together with the relevant specialists, lists all physical, medical, and dental characteristics of the body. Any elements that may possibly assist with identification of the body are photographed. The postmortem unit is therefore usually located inside the morgue, or in any other improvised location for gathering bodies if there is no morgue available.

#### The Identification Commission

The Identification Commission is made up of representatives of the ante- and postmortem units, and at minimum one forensic physician, one fingerprints specialist, one DNA expert, one odontologist, and if possible an anthropologist, police investigators in charge of the case, and the Public Prosecutor’s representative. This commission is held once the ante- and postmortem units have collected their information and after a comparative synthesis (reconciliation) has been performed. The Identification Commission declares formal identification of the individual if the data collected from the body postmortem match those collected by the antemortem unit.

The list of identifications validated by the commission shall be transmitted without delay to Public Prosecutor. The notification of death can be made by an investigator and the DVI staff that followed the family on the antemortem side, and the body may be presented if the family so wishes.

Because the DVI Police Unit works on demand and under the scope of the Investigation Service (in the case of a terrorist attack, the SDAT), it works within judicial proceedings. The antemortem and postmortem files are attached to the identification minutes generated during the commission.

## Reasons to use this methodology in response to a terrorist attack

### Expectations from other countries

The DVI methodology has been carefully developed and tested at an international level, within the framework of an INTERPOL working group that has helped it evolve for over 30 years from the experiences of each member country. France is part of this working group, and our counterparts are scrupulously applying this methodology in the case of a terrorist attack (for example, in 2004 in Atocha, or more recently in 2016 in Brussels).

Even in a disaster situation on national territory, such as that of November 2015, there are often many foreign victims (in this case 30 foreign or binational victims). Our foreign counterparts therefore expect France to identify their nationals according to the established standards. It should be noted that the DVI teams from these other countries follow the INTERPOL protocol by preparing the antemortem files, which they later send to the French DVI unit for reconciliation with the postmortem data they collected.

### Confusion created by the attacks

Even if the bodies are in good condition (face not deteriorated), the large number of them inevitably creates confusion at the scene of the attack. Personal effects can get mixed up. In the following days, many government services are involved and broadly requested to account for the victims. Coordinating all of these individuals is complex; if the procedure and protocol for the list of deceased victims are not rigorously flagged and standardized, then incorrect lists can circulate, and families can be given inaccurate information.

### Open list of missing people

Unlike in an aircraft crash situation, for example, no list of missing persons is available *ab initio*. This makes the task even more complicated because some of the missing people may be unconscious in the hospital or even safe elsewhere. A very large number of families are therefore looking for their loved ones very early on following the incident.

### Families in shock

Even if they remain unscathed, the faces of the victims are transformed by death. Following a major traumatic injury, families are not always able to recognize their loved ones. Some people might be mistaken in their recognition of victims, as was observed in November 2015 in Paris.

“Was the child I recognized a year ago my own?”

This question may come to haunt families years later. Because scientific, simple, fast, and safe identification methods are available, the responsibility for identification must not solely rest on the victims’ families.

In November 2015, among the 90 victims considered to be identified *ab initio* (those who carried identification such as an ID card and were thus presumed to be identified outside of the DVI protocol), **problematic cases from the DVI perspective have been identified:** identity swaps, incorrect death notifications, reverse presentation of bodies … namely more than 10% of the victims. The 40 victims not carrying identification documents, for whom the DVI protocol was applied, were identified with no error. In the November 2015 case, the 90 victims that were not identified through the DVI protocol, but rather presumed identified and directly presented to their next of kin, were still processed by forensic pathologists. The pathologists recorded important information such as DNA. Therefore, should any future doubt arise among the relatives regarding the individual’s identification, antemortem research could still be performed, and lead to a scientific confirmation of the victim’s identity.

**Scientific identification of all victims, even those carrying identification documents, is essential to avoid inaccurate early death notifications or presentation of bodies, as well as even incorrect burials or cremations. These events would result in major distress for the relatives, and possibly significantly prolong the search for their loved ones.**

From November 2015, the DCPJ, together with the Interministerial Cell for Helping Victims (Cellule Interministérielle d'Aide aux Victimes, hereafter CIAV), the Counter-terrorism section of the Paris Public Prosecutor’s Office (now the National Counter-terrorism Prosecutor’s Office), and the largest French morgues (especially the Paris morgue), have developed a mechanism to speed up the scientific identification process without compromising the integrity of the results. The “French style” protocol, now set up in interministerial guidelines [1], thus meets the efficiency and speed requirements posed by our modern society, while securing a methodical and scientific process of identification in the event of mass murder.

## Developments and adaptations of the DVI since the November 2015 attacks

### Division of labour and coordination of involved individuals

Numerous interministerial meetings and in-depth exchanges between the DVI Police Unit and its partners (e.g. forensic pathologists, CIAV, Counter-terrorism Prosecutor’s Office, Health Department) have enabled the various individuals interacting with the families of victims to understand the scope of each other’s missions and to coordinate together. This led to a clear procedure for families and a rewriting of the interministerial directive, “*Dealing with victims of terrorist acts*”, such that the collection of information on missing persons and death notifications took place in a much more serene and comprehensible way for families following the attack in Nice in July 2016.

### A simplified record sheet

To avoid swapping and confusing identities, no identity should be affixed to a victim when an inventory of the bodies is generated on the attack scene. The INTERPOL protocol advocates the following: each body must be singled out by a postmortem number; all victims must be registered under X (anonymous, no name associated to the body), even those carrying ID. A simplified registration form has been distributed in all judicial police departments in France, as well as in the Paris Police Prefecture. This single sheet, in French, summarizes the essential information to be recorded when collecting a body, the manner in which it must be done to secure the later DVI process, and how to attribute a postmortem number. The form is an integral part of the investigators’ methodology on an attack scene and allows for the correct listing of bodies during major crime scene findings, even if the officers involved in the collection are not DVI trained. Thus, the DVI Police Unit can efficiently take over the process when they arrive at the scene.

### Separating identification and search operations for causes of death

The search for body identifiers carried out by the DVI postmortem teams is less technical and faster than an operation for determining the cause of death performed by a forensic physician (an autopsy or a less comprehensive examination). These postmortem operations can be performed one after the other.

In November 2015, the identification-related sampling was carried out simultaneously with the search for causes of death. Postmortem teams thus depended on the pace of autopsies. Given this experience, the French DVI police unit decided to separate these two actions and to carry out the identification operations first, which can considerably reduce the identification process time.

### An Identification Commission working continuously

It is not necessary to wait until all identification operations are completed to begin the comparisons between postmortem and antemortem elements within the Identification Commission and validation of the identified persons to fill the sole list of victims and make the death notifications. This meeting can be held daily, allowing families to be notified as early as the first days following the incident.

### A fast track procedure

The abovementioned simplified record sheet indicates whether an identification document, telephone, or any other evidence suggesting the individual’s identity is found on the victim’s body. When it is the case, it is possible to carry out the postmortem operations on the bodies of these victims first (before the other bodies), and to simultaneously collect antemortem information from relatives whose identity seems linked to the victim, to quickly and scientifically validate or invalidate a suspected identity. This validation can happen as early as during the first identification commissions.

### A high degree of emphasis on secondary elements

Tattoos, scars, personal effects, and piercings are all considered “secondary” elements, which, if the bodies are not fragmented, can enable quick identification without having to wait for primary identifiers (dental, fingerprints, DNA) antemortem that may be hard to obtain. If these secondary elements are strong and/or numerous, then it is possible to provide an identification during commission on this basis, as provided in the INTERPOL guidelines [2]. However, the INTERPOL protocol provides for this as a last resort for identifying bodies where primary elements would have failed to be collected. In the interest of efficiency and speed should a terrorist attack occur on French soil, the nation takes a step back from the doctrine and offers to validate the identifications on the basis of strong secondary elements in the first reconciliation commission. This allows the most obvious identifications to be carried out in the aftermath of the attack. These identifications will then be reinforced by primary elements, but first allow for notifications to be made to families rapidly on the basis of secondary elements alone. This derogation from the INTERPOL guidelines proved to be relevant during the Nice attacks, but it is important to be extremely careful in the reconciliation commission before making such identifications.

### Background work with the largest morgues in France

Previously largely ignored by the teams of the various morgues in France, the DVI protocol was widely disseminated after the November 2015 attacks to the largest morgues in France, including those in Paris, Bordeaux, Lyon, Strasbourg, Marseille, and Lille.

During these exchanges between the DVI Police Unit and morgue teams, it was indicated that a dedicated space within the morgue was needed for the postmortem identification phase. The configuration of each morgue was studied to incorporate this requirement. In some morgues, detailed documents were developed, including a model action plan. An intervention by the DVI Police Unit at the Legal Medicine Congress in 2017 also made it possible for these recommendations to be widely disseminated to all head pathologists at morgues in France.

Finally, all morgues have needed to turn away families who came on their own to seek out loved ones after a terrorist attack. The *ad hoc* morgue should be secured so that only families duly authorized by the DVI Police Unit, following identification by the Identification Commission, can access it. An appointment is set by the DVI Police Unit and the morgue if the families wish for a presentation of the body following the notification of death at the Family Reception Center.

The morgue forbids any initiative/uncoordinated communication between the DVI Police Unit and families during the time of the crisis and redirects family phone calls to the antemortem unit.

### Interdisciplinary exercises

To test these new modes of operation, interdisciplinary exercises have been performed on numerous occasions in various places in France, which allowed for the validation of the new protocol’s effectiveness and the articulation and immediate recovery of information at the Counter-Terrorism Prosecutor’s Office, CIAV, and within morgues. Additionally, those exercises validated the effectiveness of the separation between the identification process on the one hand, and the process of looking for causes of death on the other hand.

#### Controlled communication

Only the Counter-Terrorism Prosecutor’s Office is permitted to communicate publicly about the number of victims and their identities. It does so only after the identification commission to avoid spreading inaccurate information. Updates to victim reports, also nominative, are only published by the Prosecutor’s Office once the families have been notified by the DVI Police Unit, not immediately following the identification commission or by the press to maintain privacy and respect for the families.

#### The unconscious wounded are affected by the DVI protocol

The need to apply the protocol does not stem from one individual’s state of clinical death, but because they cannot directly provide their identity. This is one of the most significant developments of the doctrine. In the chaos of mass murder, the traceability of a victim's identity is difficult to ensure. Families looking for loved ones are not aware if they have died or been hospitalized. Their loved ones could be completely unconscious in intensive care in a hospital, making the response to families all the more urgent. Identifying information should be collected from both those who died and those who are unconscious in the hospitals. Faced with the sometimes-significant injuries of unconscious people who are being treated, including head injuries, it is essential that hospital staff do not make hasty and potentially inaccurate identifications of their patients [3].

## Conclusion

In the case of a terrorist attack, the DVI INTERPOL protocol must be adhered to in the interest of families. No death notifications or body presentations should be made before validation by the Identification Commission. However, this protocol must be implemented in an efficient and quick manner to reduce the families’ unbearable waiting as much as possible. This cannot be achieved without support from the highest authorities of the State. A clear and consistent way of addressing the families is necessary. They must understand that the specialized services are making every effort to provide them with a consolidated response as soon as possible.

Through antemortem interviews and the elements they provide, the families of victims often feel they are actively involved in identifying their missing loved ones and therefore concur with the DVI’s approach. This can only be done if the State is organized to implement this in a coordinated and comprehensible manner, as it did during the Nice attacks in 2016. This was done in light of its response to the November 2015 attacks in Paris. Thus, 88 victims in Nice were identified in only 4 days without error (including four unconscious hospitalized people) in keeping with the DVI protocol and without any visual recognition by families. The protocol was also applied during the terrorist attacks in Trèbes (March 2018) and Strasbourg (December 2018).

As the repeated feedback shows (from Britain, Spain, and Belgium in particular), our foreign counterparts are meticulously applying this doctrine. The process is welcomed by the families of the victims themselves, most recently in Belgium, after their 2016 terrorist attacks. France will not remain on the outskirts of this global movement. By systematizing its application, the French DVI Police Unit has been able to make innovative adaptations to the identification protocol in the event of a terrorist attack. They also demonstrated its relevance, operational nature, and benefits to families and society in general.

## References

[1] Legifrance. Instruction interministérielle relative à la prise en charge des victimes d'actes de terrorisme. Paris (France): Legifrance; 2019. (No. l6070/SG). Available from: http://circulaire.legifrance.gouv.fr/pdf/2019/03/cir_44445.pdf. French.

[2] INTERPOL. Disaster victim identification guide. Lyon (France): INTERPOL; 2018. Available from: https://www.interpol.int/en/content/download/589/file/18Y1344EDVI_Guide.pdf

[3] Osborn D, Easthope L. Identification of the incapacitated patient in mass casualty events: an exploration of challenges, solutions, and barriers. Disaster Med Public Health Prep. 2019;13:338–344. 29956642 10.1017/dmp.2018.38

